# A Novel Index Measure of Housing-Related Risk as a Predictor of Overdose Among Young People Who Inject Drugs and Injection Networks

**DOI:** 10.1007/s11524-026-01066-2

**Published:** 2026-04-11

**Authors:** Kathleen Kristensen, Leslie D. Williams, Charlie Kaplan, Juliet Pineros, Eunhye Lee, Maggie Kaufmann, Mary Ellen Mackesy-Amiti, Basmattee Boodram

**Affiliations:** 1 Division of Community Health Sciences, University of Illinois Chicago School of Public Health, Chicago, IL USA; 2 Community Outreach Intervention Projects, University of Illinois at Chicago School of Public Health, Chicago, IL USA

**Keywords:** Housing instability, Overdose, People who inject drugs, Chicago, Negative binomial regression

## Abstract

For people who inject drugs (PWID), housing instability due to decreasing housing affordability and other factors (e.g., loss of housing due to severed relational ties, evictions due to drug use) results in added pressure on an already vulnerable population. Findings on the relationship between housing instability and overdose risk among PWID have been equivocal, in part due to inconsistent and conceptually incomplete operationalizations of housing instability. We propose a holistic, multi-indicator measure of housing instability and examine its association with drug overdose to improve understanding of its relationship to overdose. The baseline data from a network-based, longitudinal study of young PWID and their networks living in metropolitan Chicago, Illinois was analyzed to examine the relationship between a housing instability index score—consisting of five dichotomous variables assessing housing instability—and lifetime overdose count using negative binomial regression. We found a significant positive association between the housing instability score and lifetime overdose count after adjusting for 9 variables. Our results support the practical utility of a multi-indicator measure of housing instability in predicting overdose and highlight the importance of taking a holistic approach to addressing housing instability when designing interventions.

**Supplementary Information:**

The online version contains supplementary material available at 10.1007/s11524-026-01066-2.

## Background

Opioid overdose has become an increasingly salient public health crisis in the United States (U.S.) in recent years. According to the Centers for Disease Control, U.S. overdose deaths have increased in the past decade, with an increase in overdose involving synthetic opioids from 2021–2022 [1]. For people who inject drugs (PWID), this crisis is compounded by the concurrent affordable housing crisis, especially in major cities. Between 2020 and 2023, nationally, rents and home prices increased by over 20% and 35%, respectively [2], which has fostered an increase in the population of people experiencing homelessness, contributing to a 12% rise in homelessness from 2022–2023 [2].

A growing body of research has highlighted the importance of addressing structural factors, including housing, that contribute to substance use and its adverse outcomes such as overdose [3]. PWID are particularly susceptible to recurrent housing instability (i.e., lack of access to stable housing) due to ongoing barriers to maintaining and accessing housing [4]. As a result, PWID who begin to experience housing instability often remain in a state of long-term, cyclical homelessness [5]. Housing instability increases stress, depression, and anxiety, which are linked to higher overdose risk [6], and is directly associated with overdose in some studies [7–12]. Mechanisms explaining this relationship include riskier patterns of substance use among homeless PWID [4], and rushed injection in public spaces [13].

Inconsistent conceptualizations and operationalizations of housing instability are present throughout substance use research [14, 15]. In overdose research, specifically, these inconsistent conceptualizations and operationalizations have contributed to equivocal findings on the relationship between housing instability and overdose risk. Many overdose-focused research studies have measured housing instability using three main constructs—unstable housing, homelessness, and residential mobility/transience [7, 9, 11, 12, 16–19]. Among these studies, these constructs are all measured dichotomously, but operationalized in varying ways. Unstable residence has been operationalized as a self-reported measure of stable or unstable housing status as well as a dichotomized measure based on residence type (e.g., own home, couch surfing, abandoned building) [16, 17]. Similarly, homelessness has been operationalized both as a self-reported dichotomous measure (i.e., homeless/not homeless) as well as a researcher dichotomized measure based on residence type [9, 12]. Residential mobility/transience has been operationalized at individual and community levels and as both the number of moves in a period of time or the length of time in current residence (i.e., housing tenure) [7, 8].

Additionally, most extant studies examining the relationship between housing and opioid overdose have only measured a single indicator or aspect of housing instability [7, 9, 11, 12, 16–18]. Used alone, each operationalization described above is limited in fully capturing housing instability. For example, a self-reported “stable residence” often does not account for length of residency – i.e., housing tenure [20]. Likewise, housing tenure does not capture the quality of the residence, and therefore, may incorrectly identify long-term stays in unsafe environments as stable housing [21].

To address the limitations of single indicator measures of housing instability, Frederick et al. proposed that housing instability measures should be multi-dimensional (i.e., comprising multiple indicators of housing instability), an idea supported by other housing researchers assessing measures of housing instability [20, 21]. Specifically, Frederick et al. suggested a measure that includes assessments of housing type, housing tenure, legal status employment, and income [20]. Frederick et al. argued for the inclusion of indicators that affect housing stability (such as employment and legal status) as a means of capturing risk of becoming unstably housed. These indicators can help to address the inherent fluidity of housing status because, in the event that data collection occurs during a short period of time during which the respondent is relatively more stably housed than usual, these indicators can capture risk of such a respondent’s housing situation de-stabilizing. In other words, they can help to provide a more holistic picture of housing instability, rather than assuming that such a fluid and dynamic construct can be captured well cross-sectionally using indicators of current housing situations that may quickly change in the periods before and after data collection occurs [22]. Dynamic measures of housing instability that include these additional indicators of instability have therefore been called for to measure housing instability more holistically [20].

Several multidimensional approaches to measuring housing instability and insecurity have been proposed, including the Housing Instability Scale, the U.S. Department of Housing and Urban Development (HUD) Housing Insecurity Index, and the Argonne National Laboratory Housing Stability Index [23–25]. The HUD and Argonne indices are primarily designed for population-level analyses of administrative data. While the Housing Instability Scale does capture individual-level housing experiences, it does not explicitly account for criminal-legal system involvement, an important driver of housing instability among PWID. Frederick et al.’s conceptualization emphasizes individual housing experiences and structural factors like incarceration for a more comprehensive measure.

In overdose research, to some extent, researchers have heeded the call for more multi-indicator measures of housing stability by addressing both self-reported homelessness and housing tenure when measuring housing instability. A small number of studies examining housing and overdose have used measures that go beyond dichotomous operationalizations of housing status (e.g., stable/unstable, homeless/housed)[10, 19, 26], but the measures used in these studies do not account for indicators of housing instability such as employment, income, and criminal legal system involvement as called for by Frederick et. al. [20]. Therefore, there is a need for a measure of housing instability that unifies these indictors under a single index measure. Such a measure will allow researchers to understand the role of housing status more holistically as a potential predictor of overdose and other substance use-related outcomes.

Our study will address the limitations of the extant literature that has measured housing instability using varying single indicators of stability by 1) presenting a novel multi-indicator measure of housing instability and 2) examining the relationship between the new multi-indicator measure of housing instability and number of lifetime overdoses among a cohort of young PWID in the Chicago Metropolitan Area. To holistically encapsulate housing instability, the measure will include indicators of housing tenure, housing type, and self-reported homelessness, as well as the instability dimensions recommended by Frederick et al.: employment, income, and criminal legal system involvement (i.e., prior incarcerations). Unifying these indicators into a single measure of housing instability will more holistically capture the many facets of housing instability and will therefore allow for an improved understanding of its relationship with overdose.

## Methods

### Sample and Recruitment

We used baseline data from on ongoing (October 2018-March 2020) longitudinal study examining young (18–30 years old) PWID and their egocentric (personal) network members (of all ages) – including the members of their injection, sexual, and social support networks (i.e., anyone the participant had injected with, had sex with, or received money from in the past six months, respectively). Detailed study methods have previously been described [27]. In brief, to be eligible, primary participants had to (i) be between 18–30 years old, (ii) have injected drugs at least once in the past month, (iii) be able to speak English, and (iv) be a resident of the city of Chicago or surrounding suburbs during the past 12 months. Members of the egos’ injection networks were eligible for enrollment if they were (i) at least 18 years old, (ii) able to speak English, (iii) a resident of the city of Chicago or surrounding suburbs during the past 12 months, and (iv) referred to the study by an ego.

Recruitment was primarily conducted at two field sites of Community Outreach Intervention Projects (COIP), a center within University of Illinois at Chicago School of Public Health that provides services (e.g., syringe service programs—SSPs, naloxone distribution, case management, and medications for addiction treatment—MAT—services). These field sites are located in Chicago areas with higher-than-average rates of HIV, hepatitis C, sexually transmitted infections, and drug-related arrests [6]. Recruitment also included outdoor drug market areas, community organizations, and social media to reach non-SSP and suburban PWID [28]. At the baseline visit, egos were asked to recruit up to five alters, defined as people with whom they used drugs in the past six months in the same space.

### Data Collection

Data collection took place at two COIP field sites and was supplemented using a mobile van. During COVID-19, data collection shifted to Zoom to ensure safety. Study procedures were approved by the University of Illinois Chicago IRB (Protocol #2017–0388). Participants were paid hourly ($20) due to varying data collection times, averaging 2.5 h, and $50 per interview. Ego participants also earned $20 for each eligible recruited alter. All COIP services—SSP, HCV/HIV testing, counseling, case management, and linkage to care—were available to all participants and recruits, regardless of enrollment.

All COIP services (SSP, HCV/HIV testing, counseling, case management, linkage to care, etc.) were made available to all participants and to anyone recruited to learn more about the study, regardless of study enrollment status.

### Measures

#### Outcome: Lifetime Overdose Count

The outcome of interest is the number of self-reported lifetime overdoses. Overdose was defined to participants as having passed out, turned blue, or stopped breathing from using drugs. Participants were asked to report the number of times they had ever overdosed on opioids, other hard drugs, or painkillers in their lifetime.

#### Primary Covariate: Housing Instability Index

The housing instability index was the primary independent variable and consisted of the sum of five dichotomous variables—each representing a different indicator of housing instability as presented in theory by Frederick et al. —resulting in index scores from 0–5 [20]. Measures of (i) housing type and tenure, (ii) self-assessed homelessness, (iii) monthly income, (iv) employment, and (v) criminal legal system involvement were dichotomized into responses that did and did not indicate “instability.” Cutoff points for this dichotomization process were selected based on theoretical and empirical rationales as described below. Descriptive statistics for each of the variables making up the housing instability score are included in Table 1.

For the *housing type and tenure indicator*, individuals who met the definition of unstable on either the criterion for housing type or housing tenure were categorized as unstable. Housing type, as designated in the literature, is deemed unstable if the places lived in the past six months include vehicles, public transportation, abandoned buildings, shelters or welfare residences, jail, prison, a detention center, juvenile hall, or on the streets [10, 26]. For the tenure criterion of this indicator, participants who reported living in three or more different places in the previous year were categorized as unstable. This definition represents a more severe level of transience than in similar studies of PWID that used two or more as the cut-off, including one by our team [29]. These two indicators were combined as housing type and tenure are interrelated since a long-term stay in an unstable housing type (e.g., abandoned building) may be more harmful to an individual than short tenures in more stable housing (e.g., friends and family’s homes) [20]. For the *homelessness indicator*, instability was deemed if the participant answered yes to the survey question: Have you ever been homeless in the past six months? For the, *monthly income indicator,* instability was indicated if an individual reported an income of less than $999 per month in the past six months, a cutoff point that was selected due to being the lowest quartile of reported income and given that this amount is below the poverty threshold for a single individual [30]. For the *criminal legal system involvement indicator*, instability was assessed by the following survey question: How many times have you been to jail in your lifetime? Number of times in jail was used rather than ever in jail because the majority of our sample of PWID had been to jail at least once and a systematic review found that the majority of PWID experience incarceration in their lifetime [31].Based on our sample’s distribution, going to jail eight or more times (ever) was determined to be indicative of instability as this number was the cutoff for the upper quartile, and is supported by findings from the Prison and Transition Health Cohort Study in which the sample of PWID had a median of 5 prior incarcerations with the upper quartile cutoff of 9 prior incarcerations [32]. For the *employment domain*, instability was indicated if the primary source of income in the past six months was from temporary work, panhandling, recycling, state/federal benefits, public assistance, family/friends, theft, selling drugs, or sex for money as these categories did not indicate stable, legal employment or self-employment as the main source of income. Although non-traditional or illicit sources of income are not all inherently unstable, these types of employment can present challenges in obtaining housing, as they affect one’s ability to successfully apply for housing [33].

#### Other Covariates

Demographic characteristics of race/ethnicity (non-Hispanic white, non-Hispanic Black, Hispanic, and Mixed/Other), completed post high school education, gender (male, female, or transgender [all]), and age (number of years) were examined as covariates given evidence from literature supporting a relationship with overdose [34]. Other covariates listed below were examined based on theoretical support of their potential confounding of the association between housing instability and lifetime overdose count. Backloading (i.e., mixing drugs in a single syringe and then squirting into others’ syringes) and syringe sharing were included as covariates given known associations between injection risk behaviors and overdose and known associations between housing instability and increases in injection risk behaviors [35, 36]. Participants were asked how often they engaged in backloading and syringe sharing behaviors in the past six months (never, less than half the time, about half the time, more than half the time, or always). Due to low frequencies (0.9%−7.1%) the categories about half the time, more than half the time, and always, were collapsed to form one category, defined as half the time or more. Given that network characteristics have been shown to predict overdose among PWID and can theoretically be impacted by housing status, we included mean ego-alter tie strength (an indicator of relationship strength) and core network size as covariates [37]. Depression and stigma, measured using the Center for Epidemiologic Studies Depression Scale (CESD-10) and the Substance Use Stigma Mechanisms Scale (SU-SMS), respectively, were also included as covariates, as high levels of depression and stigma are known to be associated with increased overdose risk [38, 39]. The CESD-10 uses a 10-item scale to assess the frequency of depressive symptoms over the past week, with higher scores indicating greater depressive symptom burden [40]. The SU-SMS assesses three domains of substance use stigma (enacted, anticipated, and internalized) with higher scores indicating greater experiences of stigma [41]. Public versus private injection spaces were also included as covariates, as housing instability can impact one’s access to private injection spaces and public injection has been shown to be associated with increased overdose risk [42]. Participants were asked to list all injection locations in the past year and to characterize the type of place (my house, friend/relative’s house, car, public place, public transportation, abandoned building, hotel, motel, SRO, on the street, other) for each injection location. The place types of “my house” and “friend/relative’s house” were considered private and all other place types were considered public. Participants were coded as public injectors only, private injectors only, or both public and private injectors based on past-year injection locations. Residential categories (urban, suburban, transient) were also included as covariates due to known relationships between housing instability and urban areas, and literature suggesting a relationship between urban or non-urban residence and overdose [43]. Participants’ residence location was based on all listed residences for the past year and was categorized as Chicago residences only, non-Chicago residences only, or both Chicago and non-Chicago residences.

### Statistical Analysis

First, an exploration of the housing instability risk index was conducted to assess its utility. The relationship between the housing instability risk index and lifetime overdose count was then explored. Covariates were chosen based on extant literature that evidence their importance in understanding overdose outcomes among PWID. Out of the 334 participants for whom baseline data were collected, 22 who were missing data for analytic variables were excluded from the analytic sample (*N* = 312). Proportions of missingness for each variable are presented in the online supplement (Table S1). All analysis was completed in SPSS 30.0. A descriptive analysis was completed for all analytic variables. Negative binomial regression was deemed most appropriate since the outcome of interest, lifetime overdose, is a count variable, and Poisson regression was infeasible due to overdispersion in our sample (mean = 5.10, variance = 49.65). An adjusted negative binomial regression model was then generated that included the housing instability index and covariates. An additional analysis included in the supplementary material Table S2, examined an adjusted negative binomial regression model predicting lifetime overdose count, including the components of the housing instability risk index to explore whether the index measures a cumulative construct of housing instability.

## Results

### Evaluation of the Housing Instability Risk Index

Table 1 shows the descriptive statistics for the components of the housing instability risk index: housing tenure and type, self-assessed homelessness, monthly income, criminal-legal system involvement, and employment. Most participants were high-risk for housing type/tenure, self-reported homelessness, and employment, while low-risk predominated for income and criminal legal system involvement.

**Table 1:** Descriptive statistics of components in housing instability risk index

Variable	Frequency (%)	Missing
Housing Tenure and Type		0
High risk	230 (68.9%)	
Low risk	104 (31.1%)	
Self-assessed homelessness		0
High risk	214 (64.1%)	
Low risk	120 (35.9%)	
Monthly income		0
High Risk	135 (40.4%)	
Low Risk	199 (59.58%)	
Criminal-legal system Involvement		0
High Risk	154 (46.1%)	
Low Risk	180 (53.9%)	
Employment		0
High Risk	252 (75.5%)	
Low Risk	82 (24.6%)	

Table 2 shows a Cramer’s V heatmap examining the relationships between the components of the housing instability risk index and the index itself. All components were moderately to strongly associated with the index (Cramer’s V = 0.47–0.74). These associations suggest that each component of the housing instability risk index contributes meaningfully to the overall measure. The associations among the individual components of the measure were modest, except for the housing type and tenure, and self-reported homelessness components (V = 0.71). These findings indicate related but non-redundant dimensions, with some overlap between housing type/tenure and self-reported homelessness.

**Table 2:**
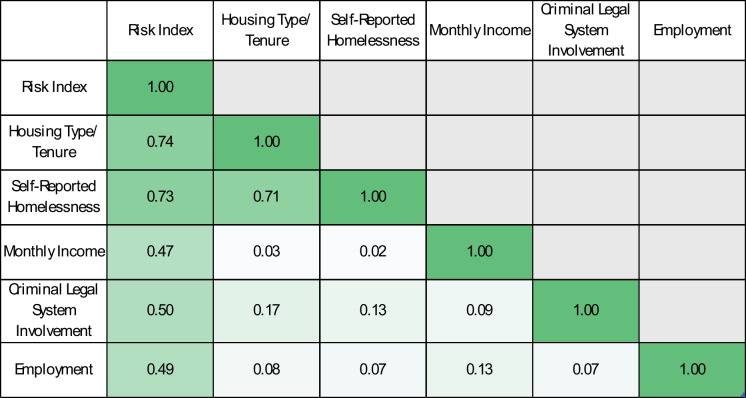
Cramer’s V heatmap showing pairwise associations among housing instability risk index components and the composite index

To further explore the utility of the housing instability risk index measure, an analysis was conducted, including the individual components of the index measure rather than the index measure in an adjusted negative binomial regression model with the outcome lifetime overdose count. The results of this analysis suggested that the index measure captured a cumulative effect beyond any single component of the measure. The results and full discussion of this analysis are included in the Supplemental Material Table S2.

### Descriptive Statistics for Variables in Regression Analysis

Table 3 reports descriptive statistics of covariates, including the primary predictor (housing instability index), injection behaviors, network characteristics, and the outcome (lifetime overdoses) for the full sample (*n* = 334). The sample was mostly non-Hispanic/Latinx white (59.9%), male (72.5%), with mean number of lifetime overdoses of 5.10 (standard deviation [s.d.] = 7.05) and the mean housing instability score was 2.95 (s.d. = 1.29) (Table 1).

**Table 3:** Study population characteristics (*N* = 334)

Variable			*N* (valid %) or mean (SD)
Male			242 (72.5%)
Female			91 (27.3%)
Transgender			1 (0.3%)
Hispanic			83 (24.9%)
Non-Hispanic White			200 (59.9%)
Non-Hispanic Black			31 (9.3%)
Mixed/Other			20 (6.0%)
Post High School Education			128 (38.3%)
Age			30.53 (7.69)
Lifetime Overdoses			5.10 (7.05)
Housing Instability Score			2.95(1.29)
Depression			2.04 (0.48)
Stigma			2.99 (0.51)
Public Injection only			135 (41.3%)
Private Injection only			74 (22.6%)
Both Private and Public Injection			118 (36.1%)
Chicago Resident Only			119 (36.2%)
Non-Chicago Resident Only			110 (30.4%)
Chicago and Non-Chicago Resident			110 (33.4%)
Core Network Size			5.65 (3.01)
Mean Ego-Alter Tie Strength			3.13 (0.55)
	Never	Less than Half the Time	Half the time or more
Backloading	203 (62.7%)	97 (29.9%)	24 (7.2%)
Syringe Sharing	186 (57.4%)	105 (32.4%)	33 (9.9%)

### Negative Binomial Regression Model

The negative binomial regression model (Table 4) used the analytic sample (*N* = 312) and included as independent variables the housing instability score and 13 covariates of interest. Variance inflation factors (VIF) were calculated to examine multicollinearity. VIF ranged from 1.05–1.87, indicating no substantial multicollinearity. Two constructs were significantly associated with lifetime overdose count: housing instability score (Wald Chi-Square = 15.10, *p* < 0.001), depression (Wald Chi-Square = 13.46, *p* < 0.001). Higher values of the housing instability score and depression were associated with a greater incidence of lifetime overdoses as indicated by incident rate ratios (IRR) displayed in Table 4.

**Table 4:** Negative binomial regression model predicting lifetime overdose (*N* = 312)

Variable	IRR^c^	Wald 95% confidence interval of IRR
**Housing Instability Score**	**1.25**	**1.12–1.39**
**Depression**	**1.72**	**1.29–2.31**
Stigma	1.26	0.96–1.65
Backloading		
1 (Never)	0.82	0.43–1.55
2 (< Half the time)	1.27	0.70–2.31
3 (> = Half the time)	1.00	
Syringe Sharing		
1 (Never)	0.70	0.40–1.23
2 (< Half the time)	0.79	0.46–1.33
3 (> = Half the time)	1.00	
Core Network Size	1.01	0.96–1.06
Mean Ego-Alter Tie Strength	1.08	0.82–1.43
Injection Setting		
Public Only	1.06	0.70–1.59
Public and Private	0.89	0.61–1.29
Private Only	1.00	
Residence Setting		
Chicago Only	0.98	0.68–1.40
Non-Chicago Only	0.99	0.68–1.43
Both Chicago and Non-Chicago	1.00	
Age	0.98	0.96–1.01
Male	1.00	0.73–1.38
Post High School Education	0.86	0.65–1.15
Race/Ethnicity		
Non-Hispanic White	1.16	0.68–1.99
Hispanic	1.14	0.63–2.06
Mixed Race/Other	0.74	0.35–1.58
Non-Hispanic Black	1.00	
	**β**	**Wald 95% Confidence Interval of β**
Dispersion Parameter	1.06	0.86–1.31

Beta estimates and standard errors are included in supplemental material Table S3

*IRR* incidence rate ratios

Bolding indicates *p*-value < 0.05

## Discussion

Our study examined a novel measure of housing instability, conceptualized as a combination of housing type, housing tenure, employment, monthly income, and criminal legal system involvement. We found a strong positive association between this housing instability score and lifetime overdose count. Additionally, depression was associated with increased lifetime overdose count To our knowledge, this study is the first to examine overdose occurrence as it relates to a holistic measure of housing instability. The significant findings indicate the usefulness of such a measure in examining housing instability among PWID.

As critics of one-indicator measures of housing instability argue, housing status is not a static condition that can simply be captured by an individual’s current residential status [20, 21]. For example, one’s financial situation or criminal legal system involvement can quickly destabilize previously stable housing. Characterizing housing status as housing instability overcomes part of this problem by considering factors that can indicate whether a person is at risk of becoming unstably housed. Conceptualizing housing status as a continuum of instability is essential to inform early intervention for PWID, as once they become unstably housed, increasing barriers may inhibit return to housing stability. However, predictive factors of stability cannot fully capture the degree to which an individual’s housing status may be changing over time. Using housing pathways that granularly track housing changes day-to-day has been suggested by some researchers, but collecting that data is time-consuming, and for the present study goes beyond the scope of available data [22].

Given the association found between the housing instability index and lifetime overdoses, intervention efforts aimed at addressing housing for PWID should focus on a holistic definition of housing instability and consider factors beyond an individual’s current residence that may be impacting their housing status. Holistic housing interventions consider both physical housing improvements and social and socioeconomic needs by providing services such as case management, substance use support, and employment assistance, and can address the complex, co-occurring processes that lead to housing instability for PWID [44, 45]. Additionally, future research on PWID should consider the use of this holistic approach to characterize housing instability to consistently capture a wider range of factors impacting housing instability and to avoid confusion in the literature that results from inconsistent use of constructs to represent housing instability.

### Limitations

The current study is limited by the cross-sectional study design. The use of baseline data does not allow for the establishment of a temporal relationship between housing instability and overdose. For PWID, current housing instability is often the result of a many-year process of destabilization, and the limitations of cross-sectional data do not allow for empirical explication of this process. Instead, a measure of recent housing instability was used to capture the extent to which participants have been impacted by this destabilization process overtime as reflected by their current housing instability. In addition, cross-sectional data cannot capture how included covariates might change over time in response to experiences of housing instability or overdose. The use of network-based recruitment for some of the participants in the study may bias results and limit generalizability, as recruitment through peer networks can result in clustering of shared characteristics and risk environments. Several of the measures making up the housing instability score in this study were also limited in their operationalization due to available data. Criminal legal system involvement was measured using data on the lifetime number of times jailed, which is limited to one specific indicator of criminal legal system involvement as the available data on prison was highly homogenous. Additionally, income was measured as a categorical variable, limiting the data to the cutoff points established by the survey question. As a result, the unstable income category included a wider range of incomes than would be ideal. Future research should include a more robust measure of criminal legal system involvement and more granular assessment of income level. The self-reported nature of these measures also presents a limitation as many of the responses relied on individuals recalling information from the past; however, the time frame for most questions was the last 6 months. Lastly, this study is limited in its generalizability as it only examines young PWID from Chicago and the surrounding suburbs. Future research should expand on the application of a holistic measure of housing instability by examining its usefulness in studying other populations of PWID.

## Conclusions

Through the examination of the relationship between a holistic index measure of housing instability and the number of lifetime overdoses, the present study addresses the need to use multi-indicator measures of housing instability when examining the association between housing status and overdose. The evaluation of the housing instability index measure and the significant association found in this study between a five-indicator housing instability score and lifetime overdoses supports the utility of such measures. These findings highlight the importance of moving beyond single-indicator conceptualizations of housing status in both research and intervention design. Future studies should test such holistic measures in broader PWID populations.

## Supplementary Information

Below is the link to the electronic supplementary material.

Supplementary file1 (DOCX 16 kb)

Supplementary file2 (DOCX 28 kb)

Supplementary file3 (DOCX 19 kb)

## Data Availability

The datasets analyzed during the current study are available from the corresponding author on reasonable request.

## Abbreviations

*CESD-10*: Center of Epidemiologic Studies Depression Scale
*COIP*: Community Outreach Intervention Project
*HCV*: Hepatitis C Virus
*HIV*: Human immunodeficiency virus
*HUD*: Housing and Urban Development
*IRR*: Incidence rate ratio
*MAT*: Medications for addiction treatment
*PWID*: People who inject drugs
*S.d.*: Standard deviation
*SRO*: Single-room occupancy
*SSP*: Syringe service program
*SU-SMS*: Substance Use Stigma Mechanisms Scale
*VIF*: Variance inflation factor
