# Supplementary material for: A Novel Index Measure of Housing-Related Risk as a Predictor of Overdose Among Young People Who Inject Drugs and Injection Networks
Source: J Urban Health. 2026 Apr 11;103(2):293–303. doi: 10.1007/s11524-026-01066-2 (PMC13235668; doi:10.1007/s11524-026-01066-2)
Supplement: Supplementary file 1 — Supplementary file1 (DOCX 16 kb) [file 11524_2026_1066_MOESM1_ESM.docx]

**Supplementary Information**

ADDITIONAL FILES

Additional File 1

File format: AdditionalFile1.docx

Title: Table S1

Description: Proportions of Missingness in Analytic Variables

Additional File 2

File format: AdditionalFile2.docx

Title: Table S2

Description: Independent Associations of Housing Instability Components with Overdose

Additional File 3

File format: AdditionalFile3.docx

Title: Table S3

Description: Negative Binomial Regression Model Predicting Lifetime Overdose with β parameter and Standard Error of β Estimates

Table S1. Proportions of Missingness for Analytic Variables

| **Variable** | **Missing Cases (%)** |
| --- | --- |
| Male | 0 |
| Race/ethnicity |  |
| Non-Hispanic White | 0 |
| Non-Hispanic Black | 0 |
| Hispanic | 0 |
| Post High School Education | 0 |
| Age | 0 |
| Lifetime Overdose | 0 |
| Housing Instability Risk Score | 0 |
| Depression | 0 |
| Stigma | 0 |
| Public Injection only | 7 (2.10%) |
| Private Injection only | 7 (2.10%) |
| Chicago Resident Only | 5 (1.50%) |
| Non-Chicago Resident Only | 5 (1.50%) |
| Mean ego-alter tie strength | 1 (0.30%) |
| Network Core Size | 1 (0.30%) |
| Backloading | 10 (3.00%) |
| Syringe Sharing | 10 (3.00%) |
