# Supplementary material for: A Novel Index Measure of Housing-Related Risk as a Predictor of Overdose Among Young People Who Inject Drugs and Injection Networks
Source: J Urban Health. 2026 Apr 11;103(2):293–303. doi: 10.1007/s11524-026-01066-2 (PMC13235668; doi:10.1007/s11524-026-01066-2)
Supplement: Supplementary file 2 — Supplementary file2 (DOCX 28 kb) [file 11524_2026_1066_MOESM2_ESM.docx]

**Table S2 Independent Associations of Housing Instability Components with Overdose**

| **Variable** | **IRR**^c^ | **Wald 95% Confidence Interval of IRR** | **β** | **Std. Error of β** |
| --- | --- | --- | --- | --- |
| Housing Type and Tenure | 1.38 | 0.87 - 2.16 | 0.32 | 0.23 |
| Self-reported Homelessness | 1.10 | 0.70 – 1.74 | 0.10 | 0.23 |
| Monthly Income | 1.23 | 0.92 – 1.64 | 0.21 | 0.15 |
| **Criminal Legal System Involvement** | **1.50**** | **1.11 – 2.02** | **0.40** | **0.15** |
| Employment | 1.10 | 0.78 – 1.54 | 0.09 | 0.18 |
| **Depression** | **1.73***** | **1.29 – 2.32** | **0.55** | **0.15** |
| Stigma | 1.25 | 0.96 – 1.65 | 0.23 | 0.14 |
| Backloading |  |  |  |  |
| 1 (Never) | 0.85 | 0.45 – 1.62 | -0.16 | 0.33 |
| 2 (<Half the time) | 1.30 | 0.71 – 2.39 | 0.27 | 0.31 |
| 3 (>=Half the time) | 1.00 |  | 0 |  |
| Syringe Sharing |  |  |  |  |
| 1 (Never) | 0.68 | 0.39 – 1.20 | -0.39 | 0.29 |
| 2 (<Half the time) | 1.30 | 0.71 – 2.39 | 0.27 | 0.31 |
| 3 (>=Half the time) | 1.00 |  | 0 |  |
| Core Network Size | 1.01 | 0.96 – 1.06 | 0.01 | 0.03 |
| Mean Ego-Alter Tie Strength | 1.11 | 0.84 – 1.48 | 0.11 | 0.15 |
| Injection Setting |  |  |  |  |
| Public Only | 1.07 | 0.71 – 1.60 | 0.07 | 0.21 |
| Public and Private | 0.92 | 0.63 – 1.34 | -0.09 | 0.19 |
| Private Only | 1.00 |  | 0 |  |
| Residence Setting |  |  |  |  |
| Chicago Only | 1.01 | 0.69 – 1.49 | 0.01 | 0.20 |
| Non-Chicago Only | 1.02 | 0.68 – 1.53 | 0.02 | 0.21 |
| Both Chicago and Non-Chicago | 1.00 |  | 0 |  |
| Age | 0.98 | 0.96 – 1.01 | -0.02 | 0.01 |
| Male | 0.94 | 0.68 – 1.32 | -0.06 | 0.17 |
| Post High School Education | 1.18 | 0.89 – 1.56 | 0.16 | 0.14 |
| Race/Ethnicity |  |  |  |  |
| Non-Hispanic White | 1.64 | 0.91 – 2.97 | 0.49 | 0.30 |
| Hispanic | 1.42 | 0.65 – 3.09 | 0.35 | 0.40 |
| Mixed Race/Other | 1.61 | 0.86 – 3.04 | 0.48 | 0.32 |
| Non-Hispanic Black | 1.00 |  | 0 |  |
|  | **β** | **Wald 95% Confidence Interval of β** |  |  |
| Dispersion Parameter | 1.05 | 0.85 – 1.29 |  |  |

Table S2 shows the results of a negative binomial regression model including all five components of the housing instability risk measure simultaneously, instead of the composite housing instability risk score. The same covariates were included in the model as those in the primary analysis model displayed in Table 4. Criminal legal system involvement was the only component of the housing instability measure that was significantly independently associated with lifetime overdose count when adjusting for all co-occurring instability factors included in the measure. The attenuation of the association between lifetime overdose count and the other four components of the index measure (housing type and tenure, self-reported homelessness, monthly income, and employment) suggests that there is overlap among these dimensions of housing instability, and the index measure captures a cumulative exposure that is not fully explained by any single indicator.

The independent association observed for criminal legal system involvement aligns with evidence that links post-incarceration release periods to elevated overdose risk (1). The observed association is consistent with established pathways by which criminal legal system involvement may increase overdose vulnerability. However, incarceration is also tightly intertwined with housing destabilization, unemployment, and income instability, suggesting that its relationship with overdose risk may operate within a broader context of housing instability. The significant association between the composite housing instability index and lifetime overdose count observed in the primary negative binomial regression model (Table 4), alongside attenuation of most individual components in the adjusted component model (Table S2), supports the interpretation that the index captures the cumulative burden of co-occurring forms of instability rather than reflecting the influence of incarceration alone.

1. Mital S, Wolff J, Carroll JJ. The relationship between incarceration history and overdose in North America: A scoping review of the evidence. Drug Alcohol Depend. 2020 Aug 1;213:108088.
