# Supplementary material for: A Novel Index Measure of Housing-Related Risk as a Predictor of Overdose Among Young People Who Inject Drugs and Injection Networks
Source: J Urban Health. 2026 Apr 11;103(2):293–303. doi: 10.1007/s11524-026-01066-2 (PMC13235668; doi:10.1007/s11524-026-01066-2)
Supplement: Supplementary file 3 — Supplementary file3 (DOCX 19 kb) [file 11524_2026_1066_MOESM3_ESM.docx]

| **Variable** | **IRR**^c^ | **Wald 95% Confidence Interval of IRR** | **β** | **Std. Error of β** |
| --- | --- | --- | --- | --- |
| **Housing Instability Score** | **1.25** | **1.12-1.39** | **0.22** | **0.06** |
| **Depression** | **1.72** | **1.29-2.31** | **0.55** | **0.15** |
| Stigma | 1.26 | 0.96-1.65 | 0.23 | 0.14 |
| Backloading |  |  |  |  |
| 1 (Never) | 0.82 | 0.43-1.55 | -0.20 | 0.33 |
| 2 (<Half the time) | 1.27 | 0.70-2.31 | 0.24 | 0.30 |
| 3 (>=Half the time) | 1.00 |  |  |  |
| Syringe Sharing |  |  |  |  |
| 1 (Never) | 0.70 | 0.40-1.23 | -0.35 | 0.29 |
| 2 (<Half the time) | 0.79 | 0.46-1.33 | -0.24 | 0.27 |
| 3 (>=Half the time) | 1.00 |  |  |  |
| Core Network Size | 1.01 | 0.96-1.06 | 0.005 | 0.03 |
| Mean Ego-Alter Tie Strength | 1.08 | 0.82-1.43 | 0.08 | 0.14 |
| Injection Setting |  |  |  |  |
| Public Only | 1.06 | 0.70-1.59 | 0.06 | 0.21 |
| Public and Private | 0.89 | 0.61-1.29 | -0.12 | 0.19 |
| Private Only | 1.00 |  |  |  |
| Residence Setting |  |  |  |  |
| Chicago Only | 0.98 | 0.68-1.40 | -0.03 | 0.18 |
| Non-Chicago Only | 0.99 | 0.68-1.43 | -0.01 | 0.19 |
| Both Chicago and Non-Chicago | 1.00 |  |  |  |
| Age | 0.98 | 0.96-1.01 | -0.02 | 0.01 |
| Male | 1.00 | 0.73-1.38 | 0.004 | 0.16 |
| Post High School Education | 0.86 | 0.65-1.15 | -0.15 | 0.14 |
| Race/Ethnicity |  |  |  |  |
| Non-Hispanic White | 1.16 | 0.68-1.99 | 0.15 | 0.28 |
| Hispanic | 1.14 | 0.63-2.06 | 0.132 | 0.30 |
| Mixed Race/Other | 0.74 | 0.35-1.58 | -0.3 | 0.39 |
| Non-Hispanic Black | 1.00 |  |  |  |
|  | **β** | **Wald 95% Confidence Interval of β** |  |  |
| Dispersion Parameter | 1.06 | 0.86-1.31 |  |  |

**Table S3. Negative Binomial Regression Model Predicting Lifetime Overdose with β parameter and Standard Error of β Estimates**
